# First Observation of Hemoglobin M Saskatoon (ß63 (E7) His>Tyr(C-T)) in the Iraqi Population

**DOI:** 10.5505/tjh.2012.71542

**Published:** 2012-10-05

**Authors:** Nejat Akar, Çiğdem Arslan, Emin Kürekçi

**Affiliations:** 1 Ankara University, School of Medicine, Pediatric Genetics Department, Ankara, Turkey; 2 Gülhane Military Medical Academy, Department of Pediatric Hematology, Ankara, Turkey

## TO THE EDITOR

Hemoglobin M Saskatoon (ß63 His>Tyr(C-T)) is a rare hemoglobin variant that was first reported in Japan, fol- lowed by the US, Indonesia, Algeria, Russia, India, and Germany [1,2,3,4,5,6,7,8]. It was also reported in combination with another variant—Hb Hamilton [9]; however, it has yet to reported in the Turkish population [10,11]. The present report describes the first observation of this variant in an 9-year-old Iraqi boy that presented with fatigue and grey-blue discoloration of the distal extremities and mucous membranes since birth. Physical examination showed cyanosis and clubbing of the fingers and toes. Complete blood count, reticulocyte count, liver and renal function tests, and abdominal ultrasound were normal. Echocardiography and angiography showed no abnormality. Blood gas analysis showed an O_2_ saturation of 91% and a methemoglobin level of 24.5%. Capillary hemoglobin electrophoresis showed hemoglobin M (Iwate or Saskatoon). Family history was unremarkable.

PCR amplification of the b-globin gene was performed using primers F:5’-GGTTGGCCAATCTACT CCCA GGAG-3’ and R:5’-GCTCACTCAGTGTGGCAAAG-3’ for exons 1 to exon 2. For exon 3 PCR amplification was performed first using the primers F:5’-CAATGTATC ATGCCTCTTT GCACC-3’ and R:5’-GAGTCAAGGCTGAGAGATACAGGA- 3’ for a 861-bp fragment, and then using the primers 5’-TGCATATAAATTGTAACTGAT-3’ and 5’-CACTGACCTCCCACA TTCCC-3’ for nested amplification. Direct automated sequencing of all amplified regions of the b-globin gene was performed using an automatic sequencer (Beckmann Coulter, USA). Two different sets of PCR reactions with forward and reverse amplification were performed. 

**Figure 1:**
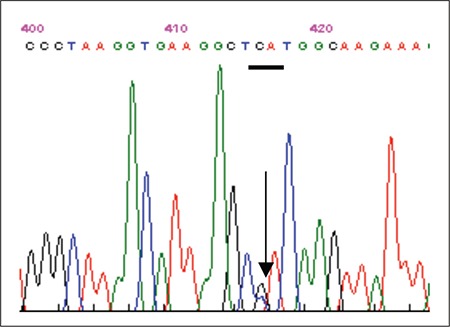
The patient’s sequencing data showing Hb M Saskatoon

The second exon amplification showed that the variant was a missense mutation at codon 63 coding for C to T transition that leads to histidine substitution by thyrosine, which was previously described as Hb M Saskatoon (Figure 1). 

There are several hemoglobin variants that cause cyanosis, of which Hb M Iwate was reported previously in the Turkish population [1,10,12]. This is the first observation of Hb M Saskatoon in an Iraqi Turkish boy. The clubbing of fingers in the propositus is an unusual finding, as patients with Hb M do not have clubbing. Despite thorough investigation, including cardiovascular procedures, we could not determine the cause of clubbing; however, Mast et al. reported 3 brothers with congenital recessive methemoglobinemia due to homozygous NADH diaphorase deficiency [13]. One of the probands had marked digital clubbing, also an unusual feature of that disease. Although HbM Saskatoon is primarily a non-hazardous disease, as HbM is susceptible to oxidative stress it is advisable to avoid oxidative drugs. 

## CONFLICT OF INTEREST STATEMENT

None of the authors has any conflicts of interest, including specific financial interests, relationships, and/or affiliations, relevant to the subject matter or materials included.
